# Automated Texture Analysis and Determination of Fibre Orientation of Heart Tissue: A Morphometric Study

**DOI:** 10.1371/journal.pone.0160735

**Published:** 2016-08-09

**Authors:** Bernhard Zach, Ernst Hofer, Martin Asslaber, Helmut Ahammer

**Affiliations:** 1 Institute of Biophysics, Centre for Physiological Medicine, Medical University of Graz, Harrachgasse 21, A-8010, Graz, Austria; 2 Institute of Pathology, Medical University of Graz, Auenbruggerplatz 25, A-8036, Graz, Austria; Washington State University, UNITED STATES

## Abstract

The human heart has a heterogeneous structure, which is characterized by different cell types and their spatial configurations. The physical structure, especially the fibre orientation and the interstitial fibrosis, determines the electrical excitation and in further consequence the contractility in macroscopic as well as in microscopic areas. Modern image processing methods and parameters could be used to describe the image content and image texture. In most cases the description of the texture is not satisfying because the fibre orientation, detected with common algorithms, is biased by elements such as fibrocytes or endothelial nuclei. The goal of this work is to figure out if cardiac tissue can be analysed and classified on a microscopic level by automated image processing methods with a focus on an accurate detection of the fibre orientation. Quantitative parameters for identification of textures of different complexity or pathological attributes inside the heart were determined. The focus was set on the detection of the fibre orientation, which was calculated on the basis of the cardiomyocytes’ nuclei. It turned out that the orientation of these nuclei corresponded with a high precision to the fibre orientation in the image plane. Additionally, these nuclei also indicated very well the inclination of the fibre.

## 1 Introduction

There are several structures in the human heart that indicate how fibres are orientated and which texture is represented by the tissue. It has been proved, that several modalities are able to detect the structure of the heart tissue [1] [2]. Examples are Diffusion Tensor Magnetic Resonance Imaging (DTMRI), Computed Tomography (CT), Ultrasonography (US) and Histology with their special image visualization methods. Although it has been shown that in-vivo imaging methods, especially DTMRI, are already able to determine fibre orientation [3], these methods are still not able to detect fibre orientation at a microscopic scale with an appropriate resolution. In particular, the cellular interstice and components of the myocytes, which could give an indication of the fibre orientation, are not visualized well enough. The size of human cardiac myocytes ranges from 80–150 μm in length and 10–35 μm in width. The nuclei of the human cardiac myocytes have an average length of 15–16 μm and a width of approximately 4–5 μm [4]. To analyse parts of the cells that give an indication of the fibre orientation, high resolution, high contrast, and low artefact images are mandatory.

Image processing methods using Hessian matrix [5] [6], Fourier transformation [7] [8] [9], fractal dimension [8], local dominant orientations [10], the grey level co-occurrence matrix [11] [12] or even second harmonic generation [13] showed good results in respect to analysing the orientation of tissue or tissue components. However, these methods just show a general orientation of the tissue image where the presence of fibrocytes, endothelial cells and cross striations remains unconsidered and bias the result of the actual fibre orientation. In addition to this, those methods just calculate a global value for the fibre orientation in the image plane but disregard perpendicular components when the nucleus axis is inclined and they do not inform about the position of individual cardiomyocytes. Mattfeldt et al. applied stereology and confocal laser scanning microscopy on fibrous structures such as polymers or glass fibres in order to test isotropy. The angular distribution of glass fibres was studied, based on pairs of registered parallel optical sections [14]. Such methods allow an unbiased estimation of the directional distribution of spatial fibre processes but finally, a fully automated statement of fibre orientation at a certain location within human tissue cannot be given.

We hypothesize that the in-plane rotational angle *φ* of individual cells and fibres as well as their inclination angle *α* in respect to the *xy*-plane can be estimated from the section contour of the nucleus in the cutting plane of a histological micrograph. This hypothesis is based on the following assumptions:

1. Nuclei are longitudinal objects in 3D and their morphology comes close to the geometry of bodies of rotation such as a cylinder or an ellipsoid.
2. Nuclei are oriented parallel to the longitudinal cell borders of myocytes and fibres.
3. The section of an obliquely cut nucleus will form an ellipse like object.
4. Micrographs show histological cross sections of nuclei with contours depending on the inclination angle *α* between the longitudinal axis *e* of the nucleus and the cutting plane.

With these assumptions it should be possible to automatically determine the angularity *φ* of fibres, myocytes and nuclei in the cutting plane as well as the inclination angle *α*. Interfering elements in the micrograph have to be removed by appropriate image processing algorithms.

The proposed method for an automatic determination of the fibre orientation in this study is named Nucleus Based Orientation (NBO) method.

## 2 Methods

### 2.1 Image acquisition

A preserved specimen of Rabbit heart from the Institute of Biophysics, Medical University of Graz was taken to obtain histological images. A Rabbit was euthanized by the professional team in the Animal Facility of the Medical University of Graz (Certified by ISO 9001: 2008 and approved by the Austrian Federal Ministry of Science, Research and Economy Approval Number: BMWF-66.010/0017-II/3b/2014) with an overdose of Propofol and Fentanyl. The heart was collected immediately after detection of animal’s death respectively cardiac arrest. This procedure was performed in accordance to the national ethical standards and regulations (§2 Abs.1 Definition of Animal Procedure, Austrian law TVG 2012 BGBL. I Nr. 114/2012 and §20: Legal Sacrification Methods TVV 2012). Correspondingly, tissue sampling is not subject to approval of an ethic committee in Austrian legislation and sacrification of animals for the sole purpose of using tissue or organs in research is per definition not an animal procedure.

**Fig 1:**
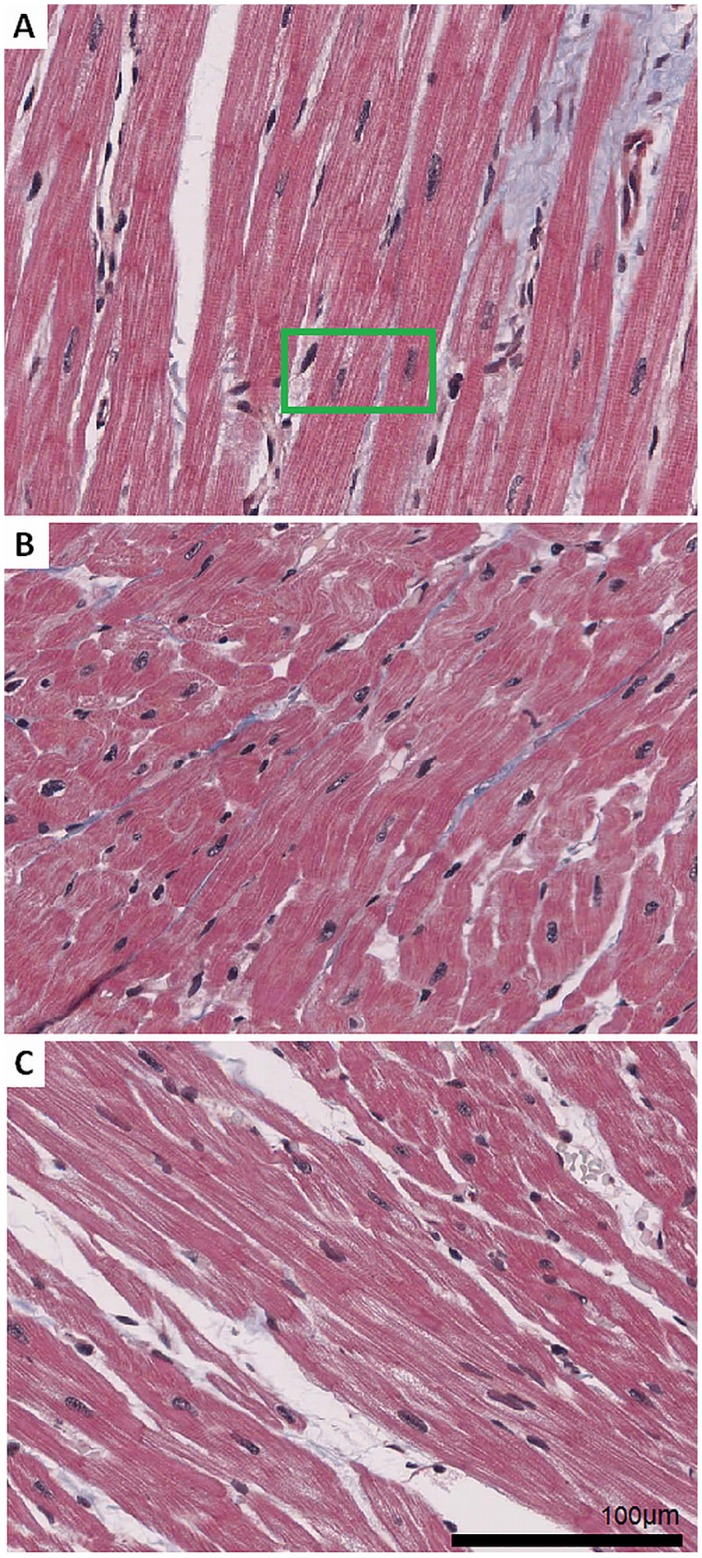
Sample images of cardiac tissue. Heart tissue slices were stained using the Trichrome (Gieson) protocol and scanned with a high resolution scanner. Sample images are cut-outs with a size of 1024 x 768 pixels. (A) Micrograph image section with fibres predominately oriented parallel to the image plane. Details of the green region of interest (ROI) are depicted in Fig 2. (B) Tissue with myocytes oriented rather oblique and perpendicular to the image plane. (C) Region with cells mixed in orientation from parallel to perpendicular to the image plane.

The heart was quickly excised and placed in modified and oxygenated Tyrode’s solution at 4–8°C for tissue preparation. The right atrium was then dissected from the isolated heart, pinned down on silicone rubber with the endocardium upwards and fixed in Carnoy’s solution, dehydrated in 96% methanol and embedded in acrylate. Slices of the atrial tissue were cut with a microtome (Polycut SM 2500E, Leica, Vienna, Austria, 2μm) and placed on glass carriers. The slices were scanned with a high resolution scanner (AperioSlide Scanner, Leica Biosystems, Nussloch, Germany). The raw data stored as SVS-file had a size of 72000 x 57000 pixels, which corresponds to a resolution of 0.279μm per pixel. The images were inspected and small sub images were cut out using the image processing software ImageScope (Aperio, Leica Biosystems, Nussloch, Germany). Three sample image sections with an image size in the range of 1024 x 623 pixels to 1232 x 786 pixels were extracted from the two scanned raw data files of Trichrome (Gieson) stained slices and exported as TIFF files. Images of these three subsections can be seen in Fig 1. We found that trichrome staining was superior to other stainings (Azan, Gomori, Hematoxylin and Eosin (HE), Oil Red O, Sudan Black) in terms of detection of nuclei.

### 2.2 Identification of tissue elements that may represent the fibre orientation

Visual inspection of the stainings showed clear differences in representing tissue structures and fibre orientation. In the tissue, fibrocytes, endothelial cells and cardiomyocytes with nuclei, intercalated discs, cell membranes, myofibrils, perinuclear sarcoplasm and cross striations were identified.

The cardiomyocyte nuclei, the intercalated discs, the cell membrane and the myofibrils were identified as potential indicators for a quantitative analysis of the tissue- and fibre orientation.

### 2.3 Reference methods for detecting fibre orientations

As a first step, reference angles *φ*_*0*_ of fibres within the cutting *xy*-plane were determined manually at ten fibres in an image using software Fiji [15] and the “Angle tool” plugin. These in-plane fibre angles served as a main reference for this study. For comparison reasons, individual in-plane angles *φ* of the nuclei, of the intercalated discs, of the cell membranes and of the myofibrils from the scanned image of the Trichrome (Gieson) stained slice were measured manually for ten different cardiomyocytes chosen randomly. Therefore, it was feasible and easy to compare the in-plane reference angles of these fibres to the individual in-plane angles of the nuclei, of the intercalated discs, of the cell membranes and the myofibrils.

In order to validate the measurements of the in-plane fibre angles and the in-plane angles of the nuclei, the measurements were performed by three different persons. For the determination of the accuracy between the in-plane reference angles and the in-plane angles of the nuclei a Mann-Whitney U test was performed.

For additional comparisons, methods using Fourier components and local gradients of an image were applied. Both methods calculate an overall in-plane angle of the texture or structure of an image, without local details of orientations or fibre directions. These two methods were applied automatically using software Fiji and the “Directionality” plugin.

### 2.4 Image pre-processing for the automatic detection of fibre orientation (NBO method)

The sample image in Fig 1A shows mostly in-plane parallel fibre orientations. The sample image in Fig 1B represents a different texture with steeper inclined fibres in reference to the cutting *xy*-plane. Finally, the sample image in Fig 1C reveals a mixture of fibres inclined from parallel to perpendicular in reference to the cutting *xy*-plane.

**Fig 2:**
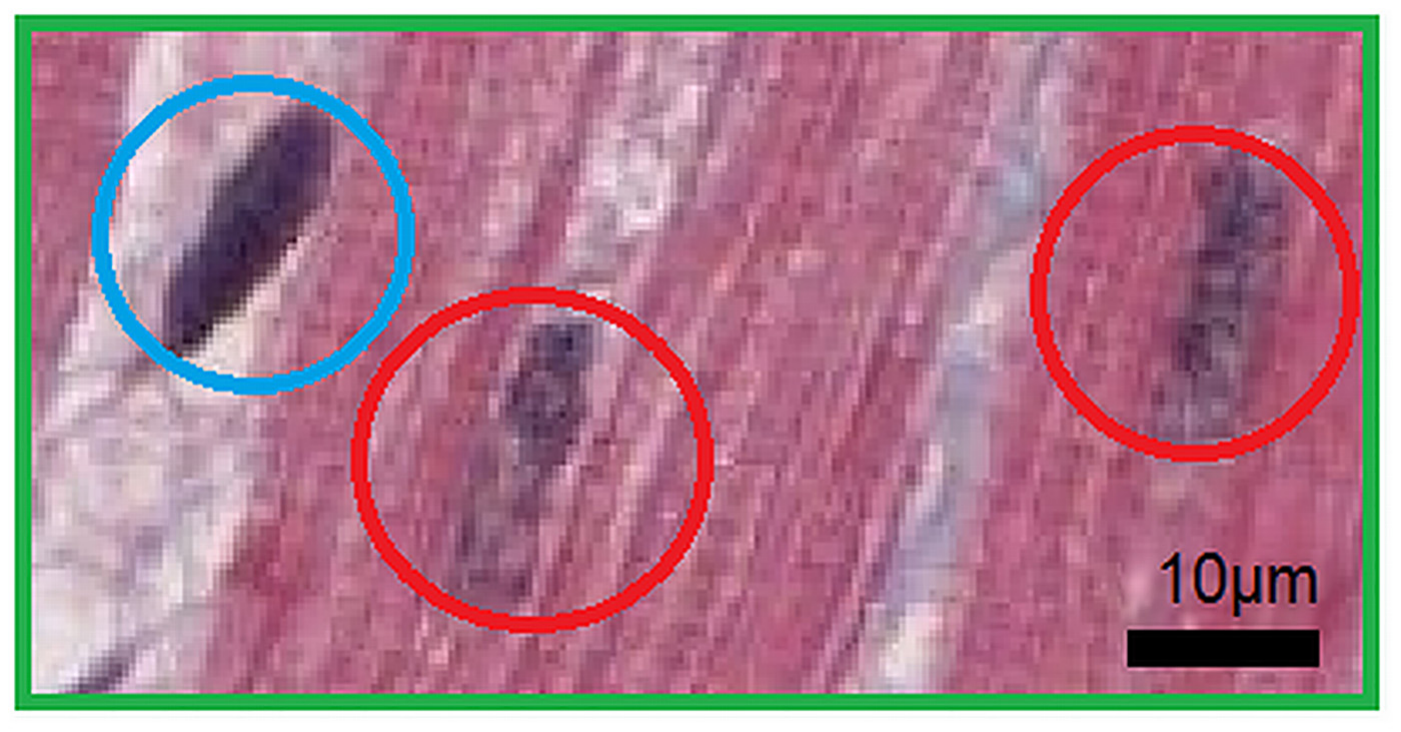
Zoomed tissue sample of image in Fig 1A (green ROI). Two nuclei of cardiomyocytes (red circles) and one nucleus of a fibrocyte on the left (blue circle) are shown. The hues of both nuclei types are very similar, whereas the chromatin arrangement and the colour saturation differ markedly.

The most difficult image processing challenge was to develop algorithms to distinguish between the nuclei of the cardiomyocytes and both the nuclei of fibrocytes and endothelial cells because the hues of the nuclei were nearly identical. However, the homogeneities of the chromatin textures were significantly different (Fig 2) and therefore, this difference has been chosen to be the main feature and criterion for the image segmentation.

**Fig 3:**
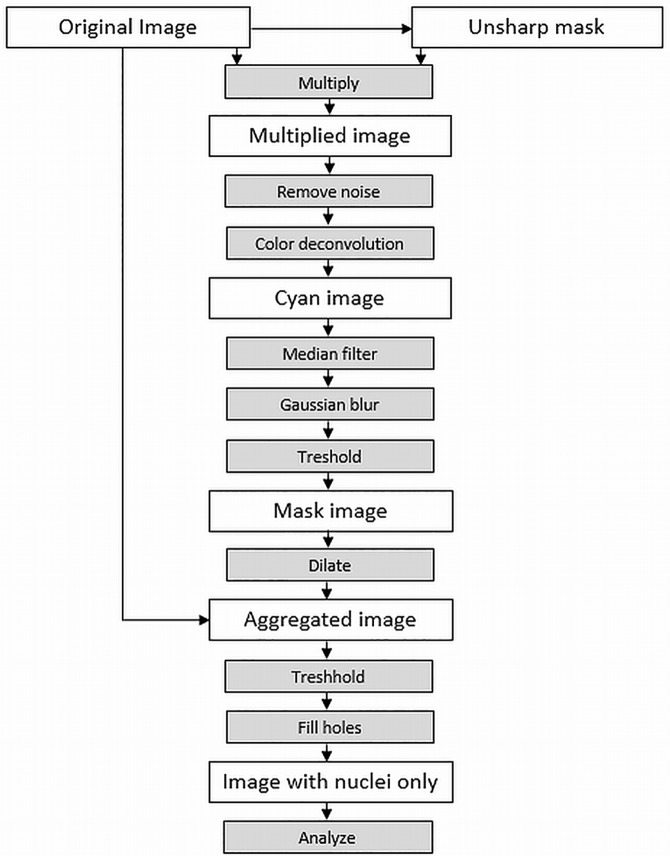
Block diagram of the image segmentation steps. In this figure, the sequence of image processing is visualized. It shows all steps that lead to an image where only segmented nuclei remain.

Following image processing steps were developed in order to identify the nuclei of the cardiomyocytes. They allow the segmentation of cardiac nuclei and to distinguish between nuclei of cardiomyocytes and nuclei of fibrocytes as well as endothelial cells. A schematic block diagram of the method can be seen in Fig 3.

Subsequent steps of the proposed image processing algorithm:

- Generation of an unsharp mask from the original image
- Multiplication of original image with this unsharp mask
- Noise removal in order to eliminate outliers
- Colour deconvolution to highlight cyan elements, which (together with black elements) represent the nuclei
- Median filtering in order to extinguish the parts of the cyan image which represent the nuclei of fibrocytes
- Gaussian blur to expand the areas of nuclei of cardiomyocytes
- Thresholding generates a binary image with black areas representing the nuclei
- Dilating the mask allows to include areas around the nuclei that may be interesting for segmentation
- Adding the original image to the dilated binary image mask so that only the areas of nuclei of cardiomyocytes remain
- Thresholding to eliminate the surrounding area of the nuclei. Slightly scattered bright areas represent the chromatin texture
- Morphologic operation to fill the bright areas within the nuclei. Black areas in the image are analysed and the results are stored in a text file

This algorithm was implemented in Fiji [15] as a macro and applied on the three sample images in Fig 1.

### 2.5 Automatic determination of fibre orientation, Nucleus Based Orientation (NBO) method

**Fig 4:**
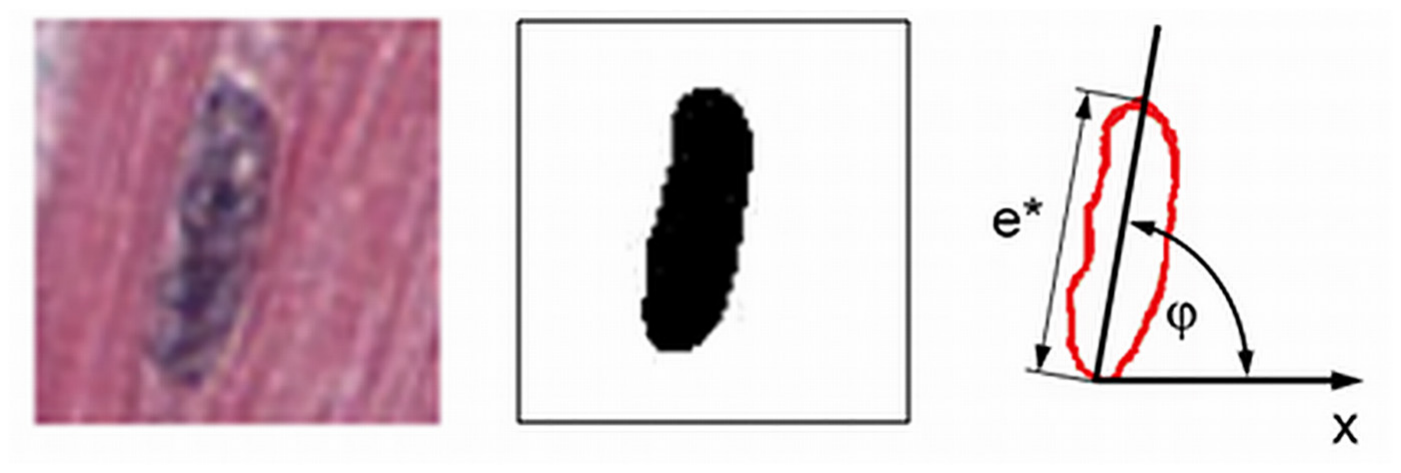
Fibre orientation based on the shape of a nucleus. The left image shows a small detail of a sample image visualizing a nucleus, the image in the middle represents the segmented nucleus as a binary image. The graph on the right depicts the scheme of determination of the angle of orientation. e* denotes the longitudinal axis of the nucleus cutting contour in the *xy*- image plane. The angle *φ* represents the in-plane angle of the nucleus with respect to the *x*-axis. Image size = 20 μm x 20 μm.

To calculate the in-plane angle *φ* of a segmented nucleus, the longitudinal axis e* of the nucleus contour and the angle *φ* of the resulting ellipse in the binary image, pre-processed as described in the previous section 2.4, was determined within Fiji using the “Analyze Particles” plugin. The angle *φ* between the major axis e* of the ellipse and the *x*-axis of the *xy*- image plane stands for the orientation of the nucleus and in further consequence for the fibre orientation in the nucleus’s neighbourhood (Fig 4).

The angle values *φ* for all nuclei as well as their positions in the *xy*-plane were computed from the image and stored in a text file which was then imported into Excel (Microsoft Office 2013). For each sample image in Fig 1 an average in-plane angle $\bar{\varphi }$ was calculated from all detected nuclei to get a general orientation of the tissue within the sample.

**Fig 5:**
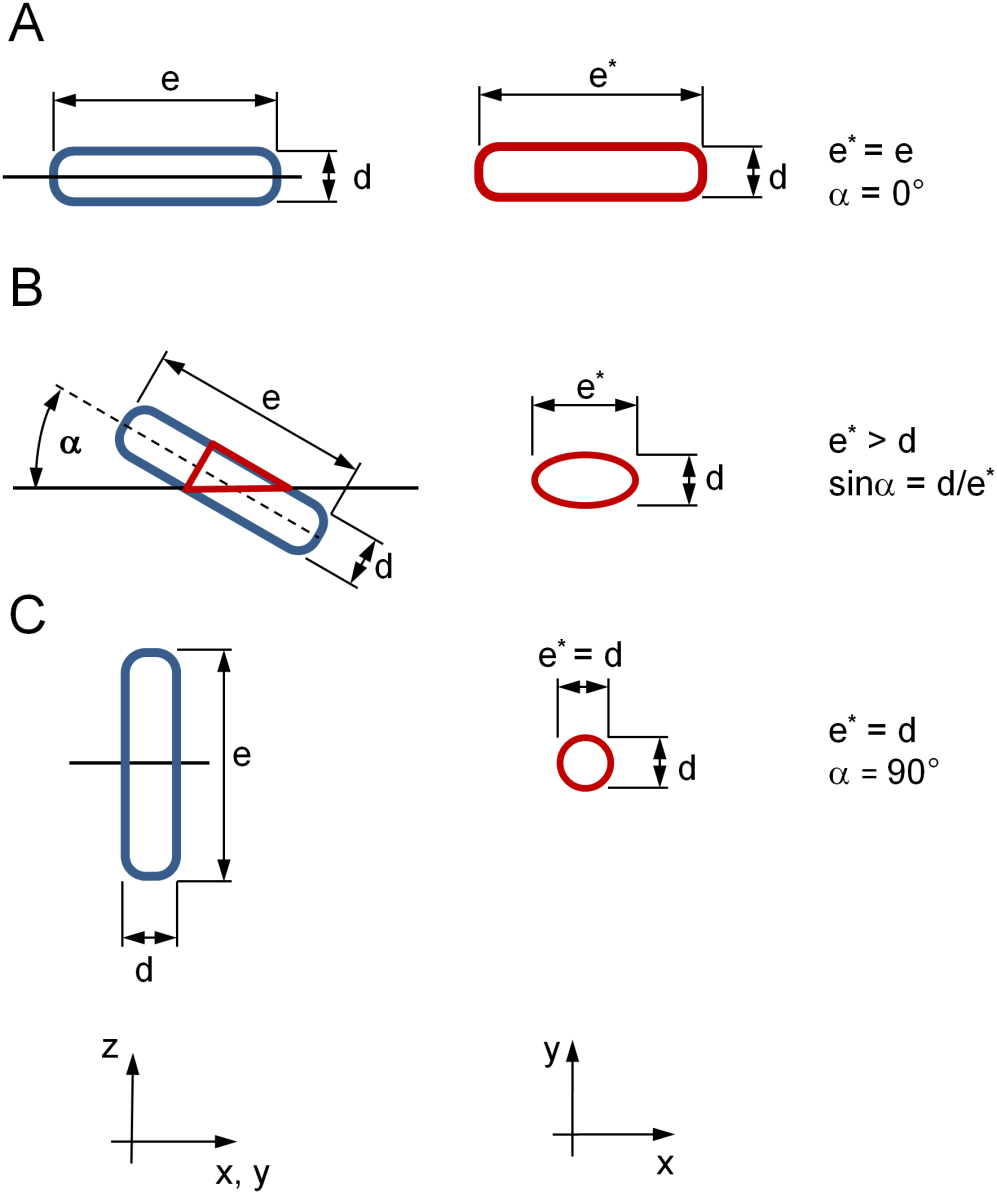
Schematic geometry of nuclei inclinations and intersections. The blue cylinders represent the lateral view of nuclei of cardiomyocytes with distinct inclination angles. The in-plane intersections of the nuclei (top view) are depicted in red. The long axis of a nucleus is denoted by *e*, the short axis is denoted by *d*. The in-plane intersection leads to a long axis denoted by e* and an unchanged short axis *d*. (A) Parallel (in-plane) nucleus *α* = 0°. (B) Inclined nucleus. The inclination angle *α* can be calculated by using a triangle. If *α* is so small that the cutting plane reaches the end faces of the cylinder, the analytical relation of *α*, *e*, e* and *d* (B) is no longer valid. If a ratio of 4:1 is assumed for the length *e* and width *d* for a nucleus, the limit for an accurate calculation of *α* is about 14°. Calculated angle values below that limit would not be valid. (C) Perpendicular nucleus *α* = 90°.

We assume that a nucleus has approximately the shape of a rotation like cylinder [16] with the length of *e*. Then the longitudinal axis e* and the perpendicular axis *d* (which corresponds to the diameter of the cylinder) from the cutting contour of the intersected nucleus can be used to calculate an estimate of fibre orientation in *z*-direction respectively the inclination angle *α* to the *xy*-image plane. This approach follows the stereological principle called method of ellipses [17] [18] and is depicted in Fig 5. From $\text{sin}\alpha =\frac{d}{e^{*}}$ the inclination angle with respect to the cutting plane *xy* can be calculated with $\alpha =arcsin\frac{d}{e^{*}}$.

Finally, the results of this NBO method were compared to the reference values (manual measurement) of the fibres and to methods computing Fourier components and local gradients of an image, as described in section 2.3.

### 2.6 Estimation of number of cardiomyocytes and a homogeneity criterion for the NBO method

In further consequence, the previously proposed and described NBO method for detecting the inclination angle *α* of a nucleus allows the calculation of additional morphometric features such as an estimate of the number of cardiomyocytes that are visible within a tissue sample.

The number of cardiomyocytes can be estimated by the proposed equation

$$N_{c}=0.9\;\frac{1}{f_{\alpha }}\frac{N_{d}}{\varepsilon },$$ (1)

where *N*_*c*_ is the number of cardiomyocytes, *N*_*d*_ is the number of detected nuclei and *ε* is the efficiency of nucleus counting, depending on the image segmentation method. The efficiency was calculated by dividing the amount of detected nuclei *N*_*d*_ by the actual number of nuclei, which was counted manually. The range of possible values for *ε* is [0, 1].

The correction factor of inclination *f*_*α*_ is the ratio of the number of visible nuclei to the number of visible cardiomyocytes in the micrograph. This correction factor depends on the inclination angle *α*. Based on the ratio of the length and the width of a nucleus compared to the length and the width of a cardiomyocyte itself, the probability to catch a nucleus of a cell is 1:6 when a the cutting plane is perpendicular to the cell axis or fibre axis and 1:2 if the cutting plane is parallel to it. Due to that fact, values of *f*_*α*_ are in the range of [1/6, 1/2], depending on the inclination angle *α* with values in the range of [90°, 0°]. Accordingly, the correction factor of inclination *f*_*α*_ is calculated by

$$f_{\alpha }=\frac{1}{6-4\left(\text{sin}\left(90-\alpha \right)\right)},$$ (2)

where *α*, according to Fig 5, is the inclination angle in degrees.

The constant 0.9 represents the fact that about 10% of the cardiomyocytes contain two nuclei [19].

Additionally, the standard deviation SD_*φ*_ of the in-plane angles *φ* of the detected nuclei and the standard deviation SD_*α*_ of the inclination angle *α* of the detected nuclei were calculated to assess the homogeneity of the fibre orientation. This value serves as a very convenient homogeneity criterion for the proposed NBO method.

## 3 Results

### 3.1 Image acquisition

Among the investigated stainings, Trichrome (Gieson) showed the best overall performance with respect to the visibility of the nuclei, the intercalated discs, the myofibrils and the connective tissue. Especially, the contrast between the nuclei, which were stained in a dark blue and the myofibrils, which were stained in pink, was very prominent.

### 3.2 Indicators for fibre orientation

The manual evaluation of the orientation of ten fibres, chosen from the image of the originally scanned slice and the orientation of their particular cell components (nuclei, intercalated discs, cell membrane, and myofibrils, as described in section 2.3) led to the following results. In order to get a value for the quality of the orientation detection, the individual differences of the detected and reference fibre angles were determined. The individual orientations and the differences to the reference measurements can be seen in Table 1.

**Table 1:** Distinct cell components and their ability to give an indication about the in-plane fibre orientation.

Fibre No.	Ref.	Nucleus		Intercalated Disc		Membrane		Myofibril	
	*φ*_0_	*φ*	Δ_*φ*_	*φ*	Δ_*φ*_	*φ*	Δ_*φ*_	*φ*	Δ_*φ*_
1	70°	63°	7°	74°	4°	69°	1°	66°	4°
2	50°	49°	1°	53°	3°	51°	1°	47°	3°
3	57°	60°	3°	49°	8°	64°	7°	60°	3°
4	27°	22°	5°	30°	3°	24°	3°	25°	2°
5	7°	8°	1°	36°	29°	4°	3°	8°	1°
6	1°	1°	0°	19°	18°	4°	3°	2°	1°
7	41°	39°	2°	25°	16°	40°	1°	38°	3°
8	79°	77°	2°	75°	3°	85°	6°	70°	9°
9	73°	72°	1°	87°	15°	69°	4°	69°	4°
10	70°	70°	0°	not visible	-	not visible	-	69°	1°
${\bar{\Delta }}_{\varphi }$			2.2°		10.9°		3.2°		3.1°

The values Δ_*φ*_ denote the difference between the in-plane reference angles of the fibres (denoted as “Ref.”) and the in-plane angles of the nuclei, the intercalated discs, the cell membrane and the myofibrils. Consequently, Δ_*φ*_ is defined by Δ_*φ*_ = |*φ* − *φ*_0_|. ${\bar{\Delta }}_{\varphi }$ is the corresponding average difference.

It turned out that the average difference ${\bar{\Delta }}_{\varphi }$ of the nuclei was only 2.2°. The average difference ${\bar{\Delta }}_{\varphi }$ of the intercalated discs showed the largest difference of 10.9°. The in-plane angles of the cell membranes showed an average difference ${\bar{\Delta }}_{\varphi }$ of 3.2° and the average difference ${\bar{\Delta }}_{\varphi }$ of the myofibrils was 3.1°. Unfortunately, particularly the intercalated discs and the cell membranes were not visible at each cardiomyocyte because neither the staining was not capable in visualizing them nor the actual part of the cell was not intersected at the required area. In addition to this, the average differences ${\bar{\Delta }}_{\varphi }$ of the intercalated discs (10.9°) and the cell membranes (3.2°) were higher than the average difference ${\bar{\Delta }}_{\varphi }$ of the cardiac nuclei (2.2°). These facts led to the conclusion that the intercalated discs and the cell membranes should not be considered for further determination of the fibre orientation by automated image processing.

The myofibrils were quite homogenous and well visible in micrographs with the Trichrome (Gieson) staining but showed an aberration around the perinuclear sarcoplasm, which partially led to an orientation in this area that differed markedly from the orientation of the myofibrils in the rest of the fibre. Furthermore, the average in-plane angle of the myofibrils showed a slightly larger difference ${\bar{\Delta }}_{\varphi }$ of the orientation (3.1°) compared to the difference ${\bar{\Delta }}_{\varphi }$ of the nuclei (2.2°).

The nucleus of the cardiomyocyte turned out to be the most accurate and most apparent element for the determination of the fibre orientation by automated image processing because the visibility of the cardiomyocytes’ nuclei within the Trichrome (Gieson) staining was very good and the average difference ${\bar{\Delta }}_{\varphi }$ of their in-plane angles compared to the reference fibre angles were very small.

The validation of the reference measurement of the in-plane fibre angles and the measurement of the in-plane angles of the nuclei by two further observers confirmed the accuracy of the manual measurement with a standard deviation of 0.03° for the fibres and a standard deviation of 0.32° for the nuclei.

A Mann-Whitney U Test led to the result that the distributions of the measured reference in-plane angles and the measured in-plane angles of the nuclei did not differ significantly (p = 0,871, n = 10).

### 3.3 Image pre-processing for the automatic detection of fibre orientation (NBO method)

**Fig 6:**
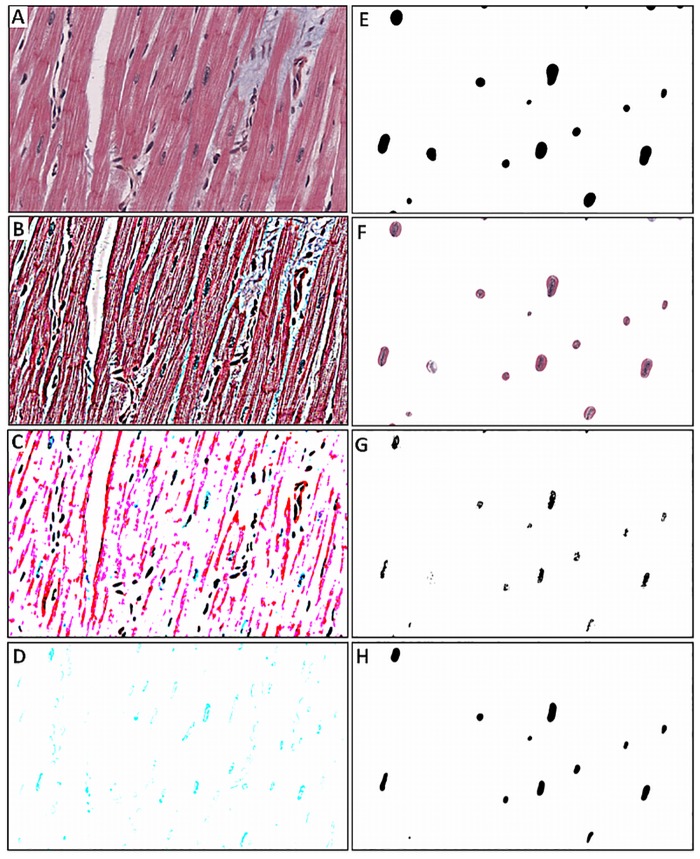
Automated image processing of sample image in Fig 1A. (A) shows the unprocessed image. (B) Generation of an unsharp mask, multiplied with (A). (C) Noise removal. (D) Colour deconvolution to extract the cyan coloured parts of the image. (E) Application of a Gaussian blur. (F) Adding (E) to (A) generates a mask around the nuclei. (G) Thresholding and removing noise and (H) Gaussian blur extracts the shape of the intersected nuclei from the generated mask.

Image processing as described above in section 2.4 was applied on three chosen sub images, taken from two scanned images of Trichrome stained tissues (Fig 1). In Fig 6 the subsequent steps of the image processing chain are depicted exemplarily, whereby Fig 6A is equivalent to the sample image in Fig 1A.

**Fig 7:**
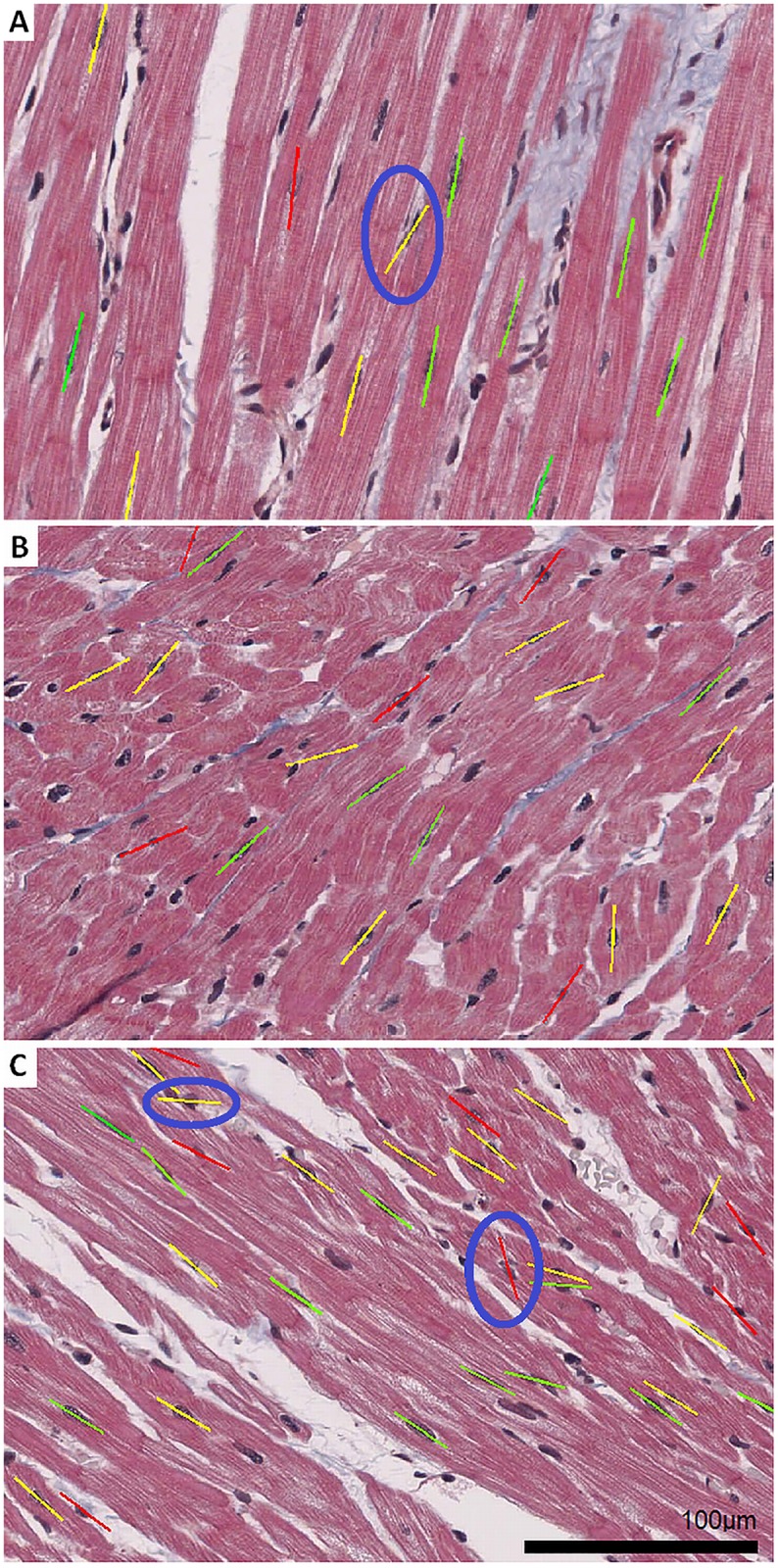
Fibre orientations depicted as dashes in the sample images corresponding to Fig 1. The nuclei were segmented and identified according to the nucleus based image processing procedure NBO (sections 2.4 and 2.5). A macro within Fiji was created which added the orientation dashes automatically to the corresponding nucleus. The orientation of a dash indicates the fibre orientation *φ* in the image plane and the colour indicates classes of inclination angles *α* of the fibres. Green represents a flat (0°-30°), yellow a moderate (30°-60°) and red a steep orientation (60°-90°). Three outliers were manually highlighted with a blue circle. (A) One nucleus of a fibrocyte in the tissue was segmented by mistake. (C) A nucleus angle was wrongly determined (yellow dash) and two nuclei were grouped together because they were located directly next to each other (red dash). The macros containing the code of the image segmentation and visualization as well as the input images and the result files were combined in ZIP files for each sample image (S1, S2 and S3 Zips).

For the sample image in Fig 1A, 12 out of 16 cardiac nuclei were segmented correctly by the automatic image segmentation algorithm. From 64 fibrocytes and endothelial cells only a single nucleus of a fibrocyte and no nucleus of an endothelial cell was segmented by mistake as a cardiomyocyte (Fig 7A). The segmentation of the sample image in Fig 1B successfully identified 19 out of 31 nuclei of cardiomyocytes and none of the 93 nuclei of fibrocytes or endothelial cells was segmented by mistake (see Fig 7B). For the sample image in Fig 1C, 28 out of 37 cardiac nuclei were correctly segmented and only 5 out of 45 nuclei from fibrocytes or endothelial cells were segmented by mistake as cardiomyocytes (Fig 7C).

The sensitivities and specificities for the image processing of the sample images in Fig 1 are listed in Table 2.

**Table 2:** Sensitivity and Specificity of the image processing method.

	Sample image Fig 1A	Sample image Fig 1B	Sample image Fig 1C
Sensitivity	75%	61.3%	75.7%
Specificity	98.4%	100%	88.9%

This table lists the sensitivity and specificity for the image processing method that was applied on the sample images in Fig 1.

### 3.4 Automatic determination of fibre orientation, Nucleus Based Orientation (NBO) method

The in-plane angles *φ* and the inclination angles *α* of the 12 segmented nuclei in sample image in Fig 1A were calculated by the Nucleus Based Orientation (NBO) method, as described in section 2.5. The average in-plane angle $\bar{\varphi }$ was 75.07° and the average inclination angle $\bar{\alpha }$ was 26° (see Table 3). The relatively low inclination is an indication for a rather flat fibre orientation related to the intersection.

**Table 3:** Comparison of several image processing methods capable to calculate the orientation of an image.

	Sample image Fig 7A			Sample image Fig 7B			Sample image Fig 7C		
Method	${\bar{\varphi }}_{0}$			${\bar{\varphi }}_{0}$			${\bar{\varphi }}_{0}$		
Reference (manual)	75.69°±5°			48.70°±13°			138.80°±9°		
	$\bar{\varphi }$	$\Delta_{\bar{\varphi }}$	$\bar{\alpha }$	$\bar{\varphi }$	$\Delta_{\bar{\varphi }}$	$\bar{\alpha }$	$\bar{\varphi }$	$\Delta_{\bar{\varphi }}$	$\bar{\alpha }$
Fourier components	77.35°±7°	1.61°	-	47.65°±18°	1.05°	-	127.25°±10°	11.55°	-
Local gradients	76.34°±14°	0.65°	-	44.15°±29°	4.55°	-	128.10°±18°	10.70°	-
Nucleus Based Orientation (NBO)	75.07°±4°	**0.62°**	26.00°	47.90°±18°	**0.80°**	37.35°	143.97°±14°	**5.17°**	35.11°

Average in-plane angles $\bar{\varphi }$, calculated by the proposed Nucleus Based Orientation (NBO) method, by using Fourier components and by local gradients compared to reference angles from manual measurements. $\bar{\varphi }$ is the average angle in the image plane, $\Delta_{\bar{\varphi }}$ is the difference of ${\bar{\varphi }}_{i}$ compared to ${\bar{\varphi }}_{0}$ of the manual measurement ($\Delta_{\bar{\varphi },i}={\bar{\varphi }}_{i}-\;{\bar{\varphi }}_{0}$, for distinct methods *i*), and $\bar{\alpha }$ is the average inclination angle.

Compared to this, the dominant in-plane orientation of the Fourier components turned out to be 77.35° and the local gradients in the image led to a dominant in-plane orientation of 76.34°. Both methods were not able to give an indication about the inclination of the tissue. In addition to this, these methods just gave a global impression of the orientation but there is no information whether there are cardiomyocytes at a certain location or not.

The average in-plane angle $\bar{\varphi }$ of the nuclei calculated with the NBO method showed the smallest deviation $\Delta_{\bar{\varphi }}$ of 0.62°, compared to the average manually measured fibres’ reference angle ${\bar{\varphi }}_{0}$ (75.69°). A comparison of the angles, calculated by the distinct methods can be found in Table 3.

The positions, sizes and angles of the nuclei have been taken from the result files and were superposed graphically to the original micrographs. This visualization can be seen in Fig 7.

By applying the NBO method on the 19 segmented nuclei in sample image in Fig 1B, an average in-plane angle $\bar{\varphi }$ of 47.90° was calculated, which can be seen in Table 3. It has to be mentioned that in sample image Fig 1B, the in-plane angles of the nuclei were not homogenous, ranging from 16.92° to 88.05°, so that a smaller detail of the tissue would have led to a more accurate average orientation. An average inclination angle $\bar{\alpha }$ of 37.35° was calculated for the 19 segmented nuclei, which confirms the visual impression that the fibres within the sample image in Fig 7B, at least in some subregions, are strongly inclined to the intersection plane, much more than the fibres in the sample image in Fig 7A. Calculation of the dominant orientation within sample image in Fig 7B by Fourier components led to a dominant orientation of 47.65° and local gradients showed a dominant orientation of 44.15°.

The average in-plane angle $\bar{\varphi }$ of the 28 segmented nuclei in sample image in Fig 1C was calculated with the NBO method to be 143.97° and from the dimensions of the intersected nuclei an average inclination angle $\bar{\alpha }$ of 35.11° was calculated. The dominant orientation in sample image in Fig 7C, calculated by Fourier components, was 127.25° and the local gradients in the image showed an orientation of 128.1° (see Table 3).

### 3.5 Summary of the morphometric investigations

By applying the automated image segmentation, as described in section 2.4 and the NBO method to detect the orientation, as described in section 2.5, the in-plane angle *φ* and the inclination angle *α* of a nucleus can be automatically calculated. Additionally, as described in section 2.6, the in-plane angles’ standard deviation (homogeneity) SD_*φ*_, the efficiency of nucleus counting *ε* and the number of cardiomyocytes *N*_*c*_ within the images were calculated. Table 4 gives an overview of actual values for the sample images in Fig 7.

**Table 4:** Overview of the morphometric investigations for sample images in Fig 7.

Morphometric values			
	Sample image Fig 7A	Sample image Fig 7B	Sample image Fig 7C
*N*_*d*_	12	19	28
*ε*	0.75	0.61	0.75
*N*_*c*_	34	78	91
$\bar{\varphi }$	75.07°	47.90°	143.97°
SD_*φ*_	4°	18°	13.8°
$\bar{\alpha }$	26°	37.35°	35.11°
SD*α*	11°	9°	13°

Images were automatically segmented and the orientation was evaluated by the NBO method. *N*_*d*_ is the number of nuclei, *ε* is the efficiency of nucleus counting, *N*_*c*_ is the number of cardiomyocytes, $\bar{\varphi }$ is the average in-plane fibre orientation, SD_*φ*_ is the standard deviation (homogeneity) of the in-plane fibre orientation, $\bar{\alpha }$ is the average inclination of the fibres and SDα is the standard deviation (homogeneity) of the inclination.

For the sample image in Fig 7A the number of nuclei *N*_*d*_ was calculated to be 12. The efficiency of nucleus counting *ε*, which depends on the segmentation method, and which is necessary to calculate the actual number of nuclei and cardiomyocytes within the image, was calculated to be 0.75. This efficiency is based on 12 detected nuclei and an actual number of 16 manually identified nuclei within the sample image in Fig 7A. The number of cardiomyocytes *N*_*c*_ was calculated to be 34 for sample image in Fig 7A. The average in-plane angle $\bar{\varphi }$, calculated with the NBO method, was 75.07°. With a standard deviation (homogeneity) SD_*φ*_ of 4° the fibres were running very homogeneously. The average inclination angle $\bar{\alpha }$ was 26°. Accordingly, the corresponding values for the sample images in Fig 1B and 1C can be seen in Table 4, too. The efficiency did not change very much, but the number of detected nuclei *N*_*n*_ and the number of cardiomyocytes *N*_*c*_ was higher since the fibre orientation led to a different appearance of the tissue. With a standard deviation SD_*φ*_ of 18° and 13.8° the in-plane fibre orientations within these images were much more inhomogeneous than the orientations in the image in Fig 7A.

## 4 Discussion

The accurately segmented and measured nucleus of a cardiomyocyte showed to be a great indicator for the automated characterization of cardiac tissue. In particular, the nucleus of a cardiomyocyte presented itself as the best element within cardiac tissue for the determination of the fibre orientation in the image plane (2D). Furthermore and exclusively, the nucleus is a unique element from which the local inclination of tissue fibres in relation to a given image plane (histological cut) can be calculated. Other methods which use all parts of the global image to detect the orientation, such as Fourier components or local dominant orientations, are also measuring elements such as fibrocytes, endothelial cells or cross-striations of cardiomyocytes which may bias the outcome. The crucial point for the automated image processing is the differentiation between the nuclei of cardiomyocytes and nuclei of cardiac fibrocytes as well as the nuclei of endothelial cells, which can show a complete different orientation than the actual orientation of the cardiomyocytes. The proposed “Nucleus Based Orientation” (NBO) method showed to be the most accurate method for the calculation of the orientation of cardiomyocytes. For all sample images, it was possible to get approximate inclination angles *α* of the segmented nuclei. The calculated inclination angles for *α* strongly confirmed the visual impressions. Furthermore, the NBO method allows calculating additional useful parameters such as the standard deviation of the in-plane angles SD_*φ*_. This parameter serves as a convenient parameter of fibre homogeneity.

However, there may be some limitations that have to be considered. The morphology of a nucleus contour and the resulting calculation of the inclination angle *α* would be the same for two inclination angles, namely *α* and 180° − *α*. A slice through a circular cylinder results in an ellipse which does not give any hint which of the two solutions for *α* is correct. In addition to this, it is obvious that small artefacts in the perimeter of detected nuclei can result in a slightly wrong calculated in-plane fibre orientation. This can happen especially for a nucleus that runs orthogonal to the cutting plane *xy*. Furthermore, due to the fact that the morphology of a nucleus comes close to the geometry of rotating bodies such as a cylinder or an ellipsoid, the calculation of the inclination angle *α* can be performed only to a certain angle *α*. If *α* is so small that the cutting plane reaches the end faces of the cylinder, the analytical relation of *α*, *e*, e* and *d* (Fig 5B) is no longer valid. If a ratio of about 4:1 is assumed for the length *e* and width *d* for a nucleus, the limit for an accurate calculation of *α* is about 14°. Calculated angle values below that limit are not valid anymore. A limitation of the image processing algorithm becomes obvious when two nuclei are located very close to each other. Then, those two nuclei are segmented together and the algorithm classifies them as one nucleus which can lead to a wrong calculation of the in-plane angle and the inclination angle for this segmented area.

Several other features of the tissue such as the amount of connective tissue or the share of cardiomyocytes could be easily identified in Trichrome stained tissue samples by simple thresholding methods. By segmenting the cardiomyocytes, other features of the tissue such as the fraction of cardiomyocytes or the ratio of the size of a cardiomyocyte to the size of its nucleus can be determined. Then, even the average size of a cardiomyocyte can be calculated if the number of cardiomyocytes in the image, based on the detected nuclei, is determined by the proposed Eq (1) in section 2.6.

With some further processing, the presented image segmentation process and the automatic NBO orientation detection method would be well predestined to allow an electro-anatomical mapping of electrical excitation data, generated by other methods [20], and the data stored in the text files. Those files contain the positions and the orientation of the nuclei and in further consequence they describe the presence, position and the orientation of cardiomyocytes within the processed images. The information of such structural parameters in histological probes is of substantial importance. It could help to classify the heterogeneity of the probe in fibre orientation which is linked closely to electrical conduction heterogeneity, an index for cardiac arrhythmia based on microstructure.

It has to be considered that the method is not validated for tissue with pathologic processes such as cardiomyopathy, atrophy or hypertrophy. Under such conditions it is likely that the NBO-method, which was developed for physiological samples, will not deliver valid results. This relates especially to image segmentation and angle calculations, since the shape of the nuclei can differ from the model-based shape under such conditions.

## Supporting Information

S1 Zip NBO method applied on sample image in Fig 1A. Contains all macros applied on sample image in Fig 1A: The input image, the segmented image, an overlay image and the results file containing the positions of the nuclei and their in-plane angles.
(ZIP)

S2 Zip NBO method applied on sample image in Fig 1B. Contains all macros applied on sample image in Fig 1B: The input image, the segmented image, an overlay image and the results file containing the positions of the nuclei and their in-plane angles.
(ZIP)

S3 Zip NBO method applied on sample image in Fig 1C. Contains all macros applied on sample image in Fig 1C: The input image, the segmented image, an overlay image and the results file containing the positions of the nuclei and their in-plane angles.
(ZIP)
