# Supplementary figures and images for: Automated Texture Analysis and Determination of Fibre Orientation of Heart Tissue: A Morphometric Study
Source: PLoS One. 2016 Aug 9;11(8):e0160735. doi: 10.1371/journal.pone.0160735 (PMC4978441; doi:10.1371/journal.pone.0160735)

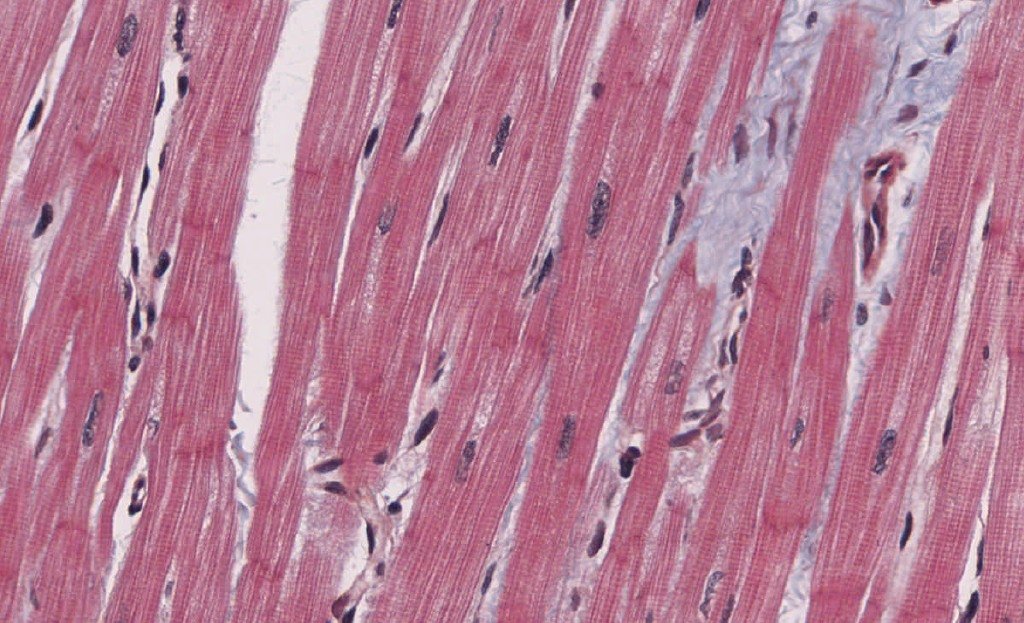

Supplement: S1 Zip — Contains all macros applied on sample image in Fig 1A: The input image, the segmented image, an overlay image and the results file containing the positions of the nuclei and their in-plane angles. (ZIP) [file pone.0160735.s001.zip › Sample A/Fig_A.jpg]

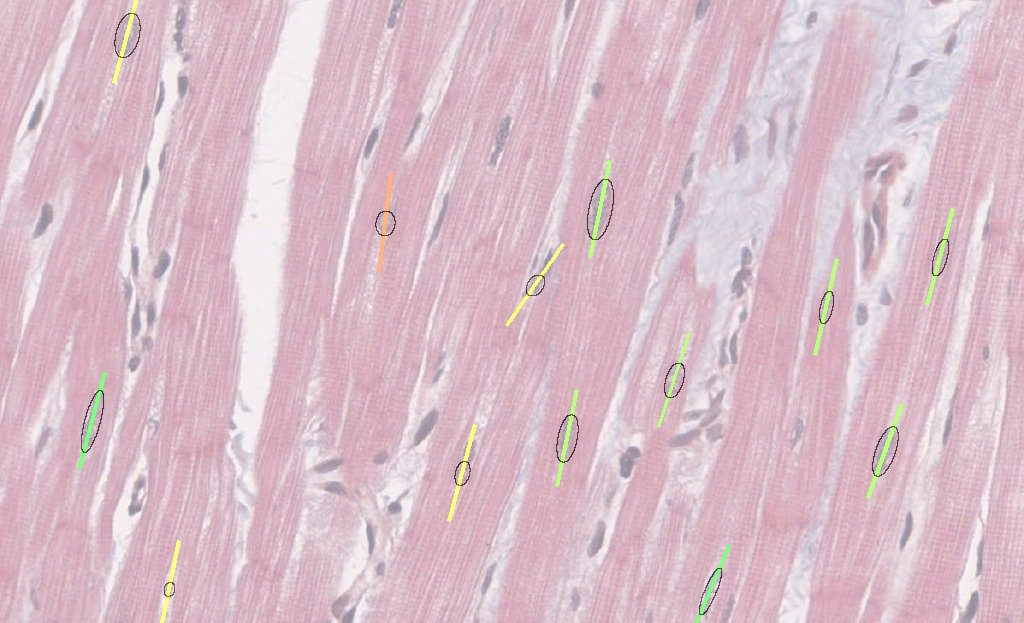

Supplement: S1 Zip — Contains all macros applied on sample image in Fig 1A: The input image, the segmented image, an overlay image and the results file containing the positions of the nuclei and their in-plane angles. (ZIP) [file pone.0160735.s001.zip › Sample A/Fig_C.jpg]

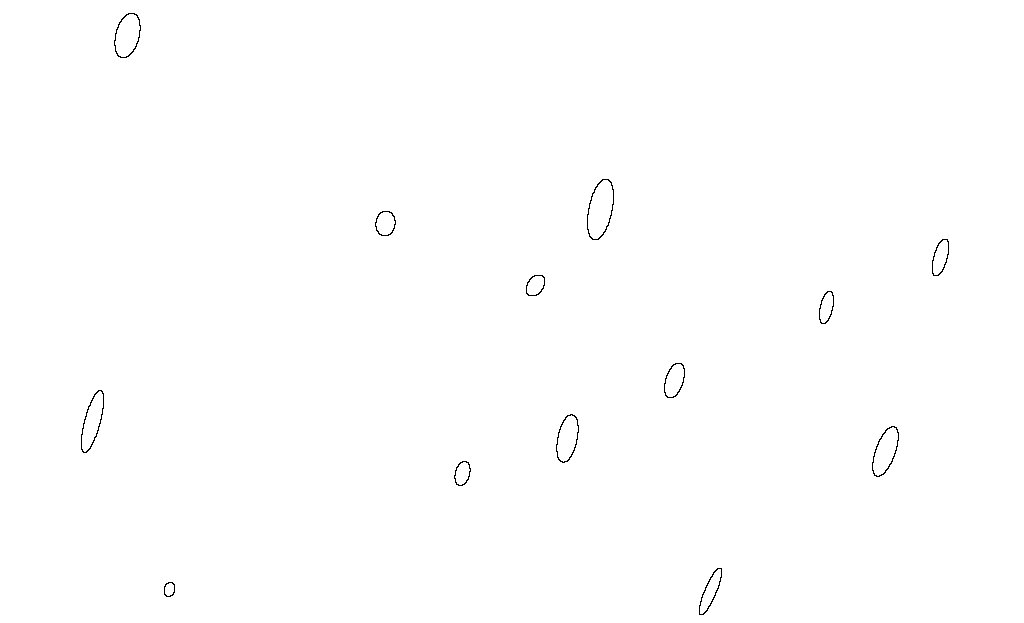

Supplement: S1 Zip — Contains all macros applied on sample image in Fig 1A: The input image, the segmented image, an overlay image and the results file containing the positions of the nuclei and their in-plane angles. (ZIP) [file pone.0160735.s001.zip › Sample A/Fig_B.jpg]

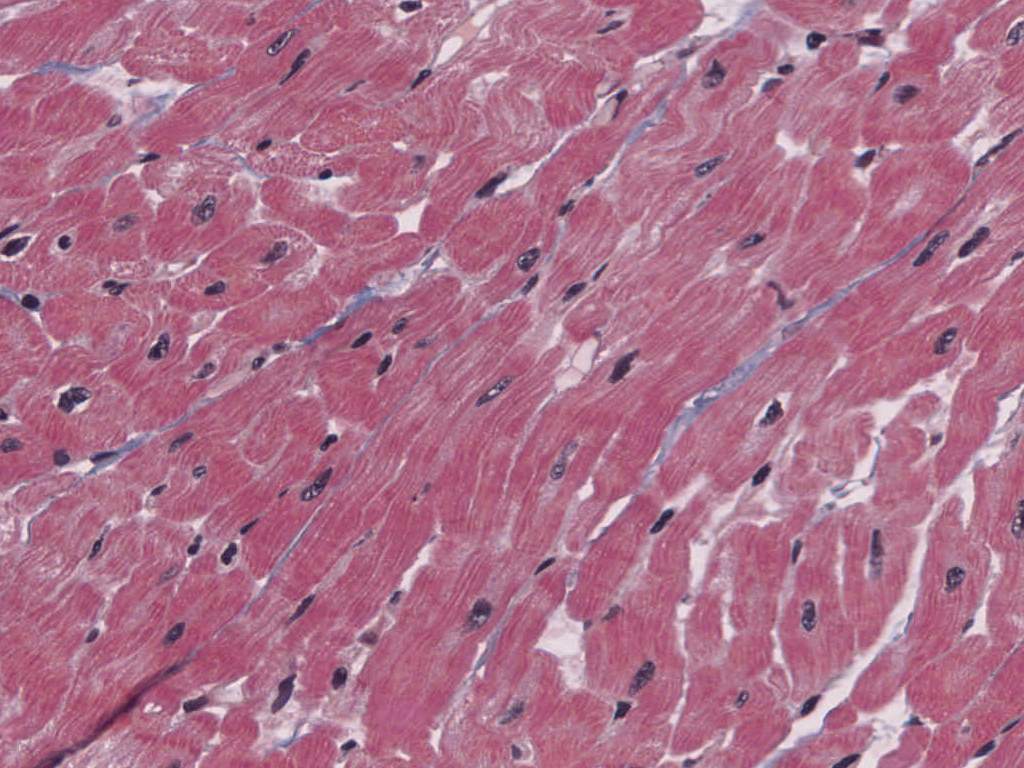

Supplement: S2 Zip — Contains all macros applied on sample image in Fig 1B: The input image, the segmented image, an overlay image and the results file containing the positions of the nuclei and their in-plane angles. (ZIP) [file pone.0160735.s002.zip › Sample B/Fig_A.jpg]

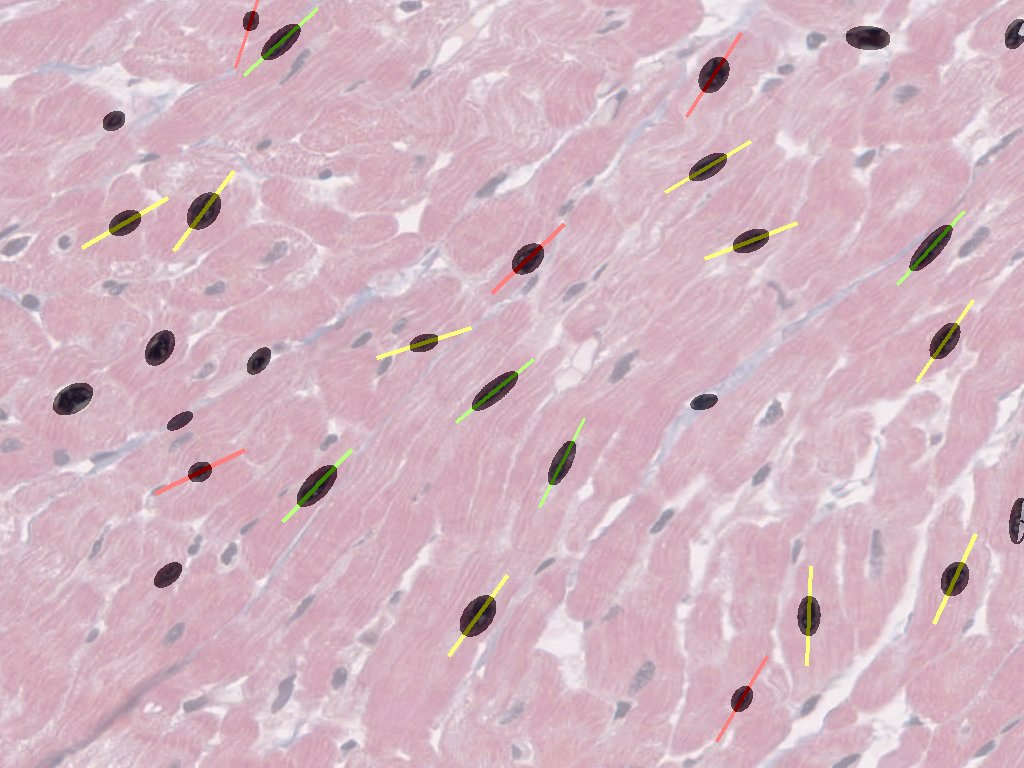

Supplement: S2 Zip — Contains all macros applied on sample image in Fig 1B: The input image, the segmented image, an overlay image and the results file containing the positions of the nuclei and their in-plane angles. (ZIP) [file pone.0160735.s002.zip › Sample B/Fig_C.jpg]

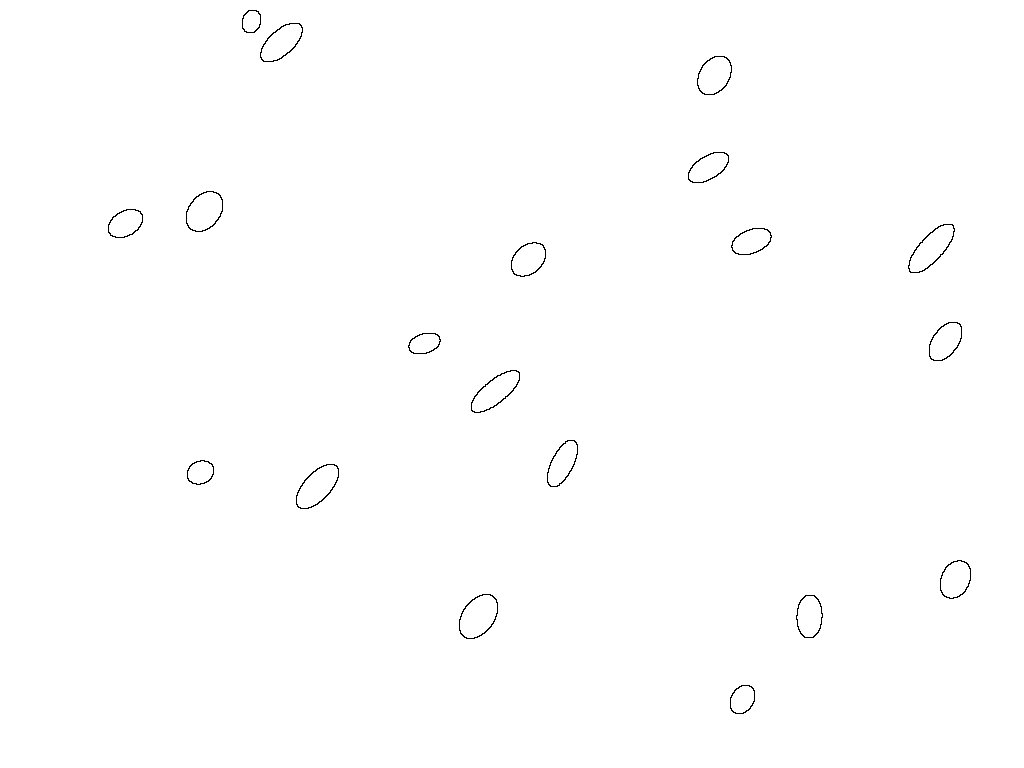

Supplement: S2 Zip — Contains all macros applied on sample image in Fig 1B: The input image, the segmented image, an overlay image and the results file containing the positions of the nuclei and their in-plane angles. (ZIP) [file pone.0160735.s002.zip › Sample B/Fig_B.jpg]

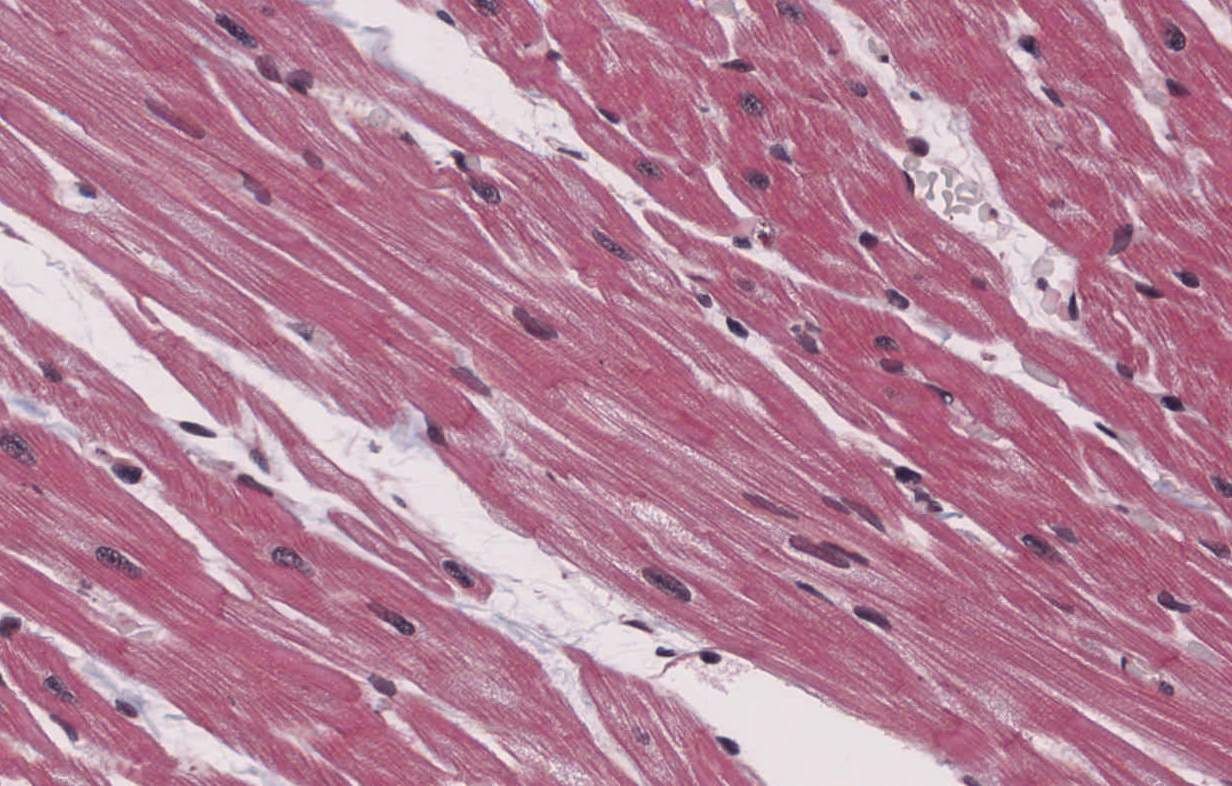

Supplement: S3 Zip — Contains all macros applied on sample image in Fig 1C: The input image, the segmented image, an overlay image and the results file containing the positions of the nuclei and their in-plane angles. (ZIP) [file pone.0160735.s003.zip › Sample C/Fig_A.jpg]

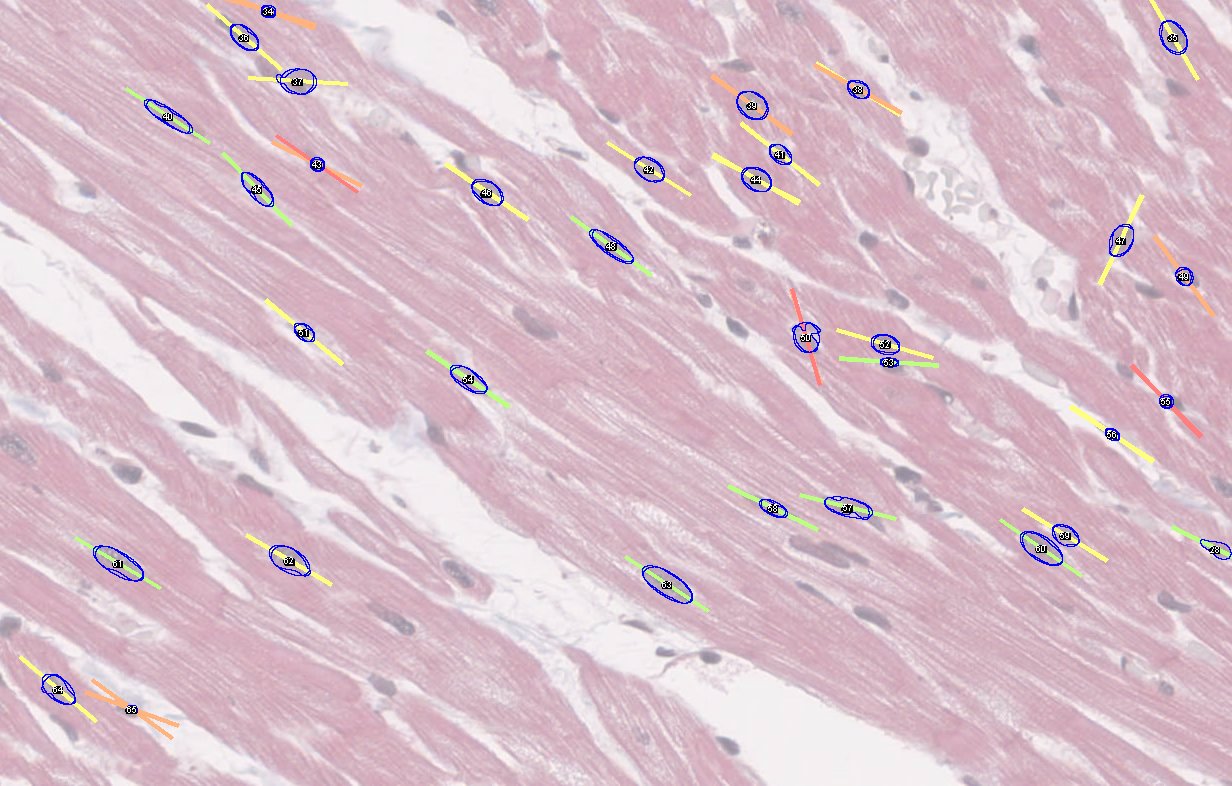

Supplement: S3 Zip — Contains all macros applied on sample image in Fig 1C: The input image, the segmented image, an overlay image and the results file containing the positions of the nuclei and their in-plane angles. (ZIP) [file pone.0160735.s003.zip › Sample C/Fig_C.jpg]

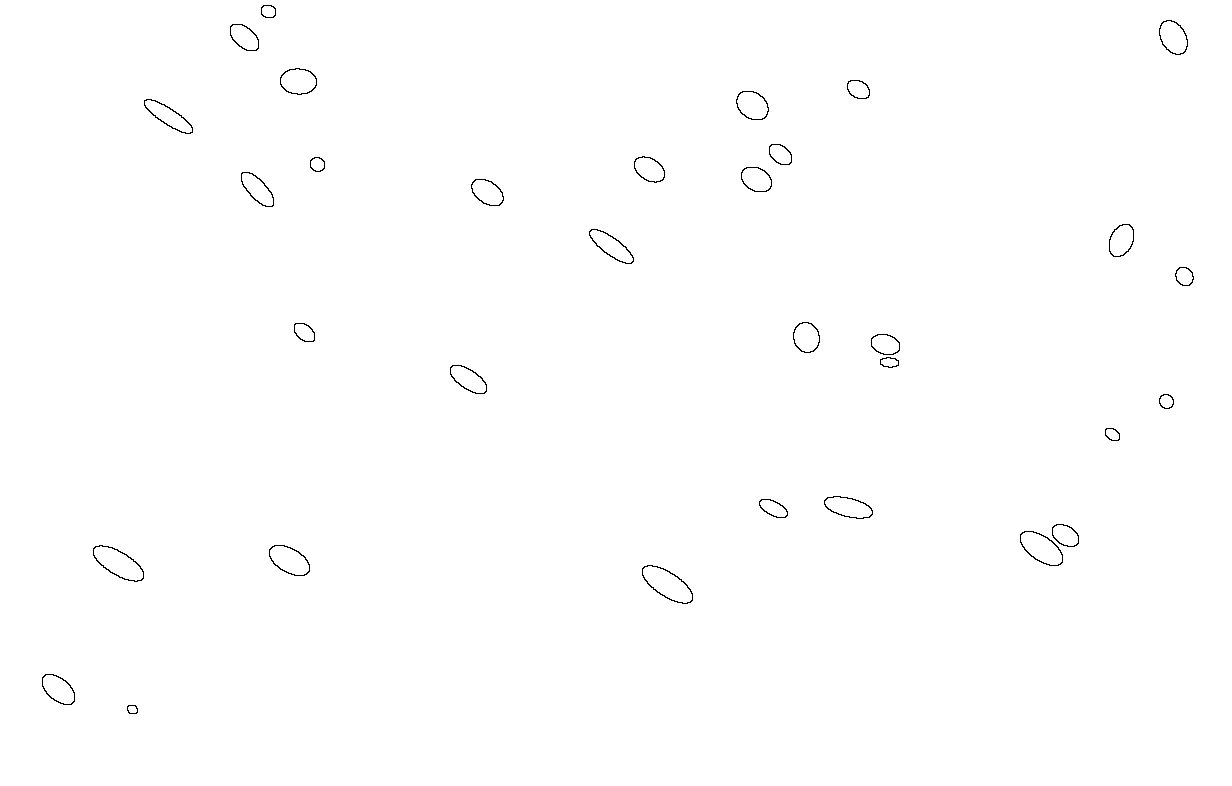

Supplement: S3 Zip — Contains all macros applied on sample image in Fig 1C: The input image, the segmented image, an overlay image and the results file containing the positions of the nuclei and their in-plane angles. (ZIP) [file pone.0160735.s003.zip › Sample C/Fig_B.jpg]
